# Editorial: The Physical Chemistry of Organic-Inorganic Interfaces as a Key to Understanding Hybrid Nanomaterials

**DOI:** 10.3389/fchem.2022.952268

**Published:** 2022-07-19

**Authors:** Álvaro W. Mombrú, Ricardo Faccio, Mariano Romero, Alfredo Juan, André A. Pasa

**Affiliations:** ^1^ Centro NanoMat/CryssMat and Física, DETEMA, Facultad de Química, Universidad de la República (UdelaR), Montevideo, Uruguay; ^2^ PLAPIQUI, Universidad Nacional del Sur - CONICET, Camino La Carrindanga, Bahía Blanca, Argentina; ^3^ Laboratório de Filmes Finos e Superfícies (LFFS), Universidade Federal de Santa Catarina, Florianopolis, Brazi

**Keywords:** organic-inorganic interfaces, physical chemistry, hybrid nanomaterials, surfaces, applications

Research on organic-inorganic hybrid materials has been developing and increasing since the 1990s. The reason for this increase is the combination of the properties of each of the components, with very different characteristics. The crystalline structures and composition, which emerge directly from the electronic structures, play fundamental roles, and the interaction between both types of materials, creates zones with defined local properties. Over the years, the above has been used for various purposes. There have been many advances in the preparation and characterization of hybrid organic-inorganic materials and it is widely accepted that many magnetic, ionic, electronic, and optoelectronic properties can be drastically enhanced compared to their isolated counterparts. However, there is still an outstanding need to understand the mechanisms involved in the physical chemistry processes at these complex organic-inorganic interfaces.

In “Factors influencing the interactions in gelatin/hydroxyapatite hybrid Materials” (Z. Zhang et al.) the focus is on the improvement of scaffolds by using the standard material for this purpose, hydroxyapatite, and trying to take advantage of the good properties of gelatin, which has led to a steady increase in interest from research groups due to the mechanical properties and the versatility they offer, which has also brought huge attention to hydrogels. The combination of both materials plays an analog role as cement and steel for the construction of strong buildings. Both materials contribute to creating a hybrid scaffold with mechanical properties improvements. This Research Topic presents a thorough study on the nucleation and growth of hydroxyapatite crystals is presented, giving important information on the state-of-the-art in this field.

In “Aramid-zirconia nanocomposite coating with excellent corrosion protection of stainless steel in saline media” A. A. Nazeer et al. focus on another field of opportunities for this kind of system. Through the preparation of nanocomposites and the deposition as coatings. In particular, the authors use this methodology to provide stainless steel protection against saline media. Microstructure and crystal structure are key for the achievement of this task, where embedding zirconia in the aramid polymer proved to enhance the intrinsic barrier characteristics of pure aramid.

Another application where the presence of hybrid organic-inorganic systems is important is the development of perovskite solar cells. Contributing to this topic, the article “Terahertz Wave Absorption Property of all Mixed Organic-Inorganic Hybrid Perovskite Thin Film MA (Sn,Pb) (Br,I)_3_ Fabricated by Sequential Vacuum Evaporation Method” I. Maeng et al. presents a work in thin films prepared by sequenced vacuum evaporation method in the CH_3_NH_3_(Pb,Sn) (Br,I)_3_, perovskite systems where the replacement of Pb for Sn and I for Br, in this generic composition is studied through time-domain spectroscopy. Authors scanned in different annealing conditions where main crystal structure parameters and crystalline systems are reported and concluded the feasibility of such systems to find applications in the THz region, for example as sensors and modulating devices.

Finally, in their mini-review on “Hybrid organic-inorganic materials and interfaces with mixed ionic-electronic transport properties: advances in experimental and theoretical approaches,” Romero et al. discuss the state-of-the-art of mixed ionic-electronic transport properties of hybrid organic-inorganic materials, mainly focusing on the phenomena occurring in the interface, both from experimental points of view and in theoretically simulations. They emphasize ionic and electronic conducting polymers interacting with semiconducting oxides such as ZnO, TiO_2_, SiO_2,_ and also on In-doped SnO_2_, ITO, which is widely used as a conducting film for glasses and other substrates.

These four contributions are good examples of the variety of applications that can be explored when performing research in the field of hybrid organic-inorganic materials. Areas such as energy conversion and storage, corrosion, and mechanical reinforcement of tissue replacement scaffolds or sensors, are just representative examples of the different ways in which this subject can offer very interesting solutions.

